# A Simple Strategy for Development of Single Nucleotide Polymorphisms from Non-Model Species and Its Application in *Panax*

**DOI:** 10.3390/ijms141224581

**Published:** 2013-12-17

**Authors:** Ming Rui Li, Xin Feng Wang, Cui Zhang, Hua Ying Wang, Feng Xue Shi, Hong Xing Xiao, Lin Feng Li

**Affiliations:** Key Laboratory of Molecular Epigenetics of Ministry of Education, Northeast Normal University, Changchun 130024, China; E-Mails: limr145@nenu.edu.cn (M.R.L.); wangxf571@nenu.edu.cn (X.F.W.); zhangc034@nenu.edu.cn (C.Z.); wanghy609@nenu.edu.cn (H.Y.W.); shifx816@nenu.edu.cn (F.X.S.)

**Keywords:** conservation genetics, *Panax ginseng*, *Panax quinquefolium*, single copy nuclear gene

## Abstract

Single nucleotide polymorphisms (SNPs) are widely employed in the studies of population genetics, molecular breeding and conservation genetics. In this study, we explored a simple route to develop SNPs from non-model species based on screening the library of single copy nuclear genes (SCNGs). Through application of this strategy in *Panax*, we identified 160 and 171 SNPs from *P. quinquefolium* and *P. ginseng*, respectively. Our results demonstrated that both *P. ginseng* and *P. quinquefolium* possessed a high level of nucleotide diversity. The number of haplotype per locus ranged from 1 to 12 for *P. ginseng* and from 1 to 9 for *P. quinquefolium*, respectively. The nucleotide diversity of total sites (π_T_) varied between 0.000 and 0.023 for *P. ginseng* and 0.000 and 0.035 for *P. quinquefolium*, respectively. These findings suggested that this approach is well suited for SNP discovery in non-model organisms and is easily employed in standard genetics laboratory studies.

## 1. Introduction

Detection and assessing the genetic variations of a given species is one of the fundamental issues in biology. Since Mendel initially developed the phenotype-based genetic markers in his experiments, the identification and employment of genetic markers have made great progress in the past decades [1]. Specifically, a series of molecular markers have been explored due to the advances in molecular technologies. For example, restriction fragment length polymorphism (RFLP) is the first DNA marker that provides an efficient molecular tool to evaluate the genetic variation of a species [2]. This hybridization-based technique is widely utilized to detect DNA polymorphisms because of its relatively high polymorphic, co-dominantly inherited and highly reproducible. In addition, the development of polymerase chain reaction (PCR)-based molecular markers, such as random amplified polymorphic DNA (RAPD), amplified fragment length polymorphism (AFLP) and microsatellite, also supply an array of approaches that yield a large number of genetic variations in different organisms [3,4]. For instance, the microsatellite markers are broadly employed as a reliable DNA marker for multiple purposes across a wide range of species, including QTL tagging, population genetics, molecular breeding and phylogenetic analysis [5–7].

In recent years, however, the availability of abundant genetic resources for numerous organisms is contributing to a transition to the use of single nucleotide polymorphisms (SNPs) [8]. In particular, recent progresses in the cost and accuracy of high throughput sequencing technologies are revolutionizing the opportunities for producing genetic resources in different organisms [9]. For example, Geraldes *et al.* [10] have identified 0.5 million putative SNPs in 26,595 genes of the model species black cottonwood (*Populus trichocarpa*) using high-throughput sequencing technology. Similarly, Howe *et al.* [11] have also characterized 278,979 unique SNPs from the non-model species *Pseudotsuga menziesii* through screening of a reference transcriptome. Notably, although the next generation sequencing platforms have generated a large numbers of SNPs in both model and non-model organisms, some of these DNA polymorphisms are distributed in the duplicate regions of the genome (*i.e.*, different members of the same gene families) that might result in the paralogous sequence variants (PSVs) and eventually limit the utilization of SNPs. Therefore, there is an urgent need to develop reliable SNPs from single copy nuclear genes (SCNGs) that could be used for applications such as molecular phylogenetics and genetic mapping. To this end, we explored a simple and straightforward approach to characterize SNPs from *Panax ginseng* C.A. Meyer and *P. quinquefolium* L. by screening the constructed library of Arabidopsis SCNGs. *Panax* L. (Araliaceae), commonly known as ginseng, is a medicinally important genus in the Orient and includes 18 species with 16 from eastern Asia and two from eastern North America [12,13]. *P. ginseng* is one of the highest valued medicinal species within *Panax*. Although *P. ginseng* was widely distributed in Russia, Korea and China at the beginning of 20th century, there exists only a few individuals in natural environments due to the over exploitation of wild resources and the destruction of natural habitats [14,15]. To date, *P. ginseng* has been listed as a rare and endangered plant in China [16]. Similarly, *P. quinquefolium* L. (American ginseng) is also a medicinal plant which is native to North America and widely cultivated in China [17]. Results from molecular phylogenetic analyses revealed that *P. ginseng* and *P. quinquefolium* are most closely related species within this genus [12,13]. To explore reliable SNPs from *P. ginseng* and *P. quinquefolium*, we developed 16 single copy nuclear genes (SCNGs) from the *Panax* dbEST of GenBank (http://www.ncbi.nlm.nih.gov/dbEST/index.html) (1 October 2012) [18]. These SCNGs may provide a series of useful molecular markers for future studies of conservation genetics.

## 2. Results and Discussion

### 2.1. Development of SNPs from SCNG Library

The predominant type of molecular genetic marker has changed substantially over the past decades [8]. To date, SNP markers have come to prominence due to the abundant polymorphism in genomes, low-scoring error rates and relative ease of calibration among laboratories [9]. Specifically, with the advances in DNA sequencing technologies, SNP markers have contributed greatly to the genetic studies of model organisms. A large numbers of SNPs were retrieved from Arabidopsis, Oryza and Populus via the employment of high throughput DNA sequencing platforms [19–22]. Nonetheless, the application of SNPs in non-model species lagged behind because of the limitation of marker development and the existence of PSVs [23]. Although the strategies of transcriptome and reduced representation genomic libraries sequencing also generated a numbers of SNPs from different non-model organisms, utilization of these SNP discovery approaches as standard tools in non-model species remain challenging thus far [24–27]. The main stumbling block hindering wide adoption of SNPs in non-model organisms is that these next generation sequencing technology-based approaches are too expensive for the population level analysis, in particular to these studies with large sample size, because it is sometimes impossible to assemble all the short reads without a reference genome. In addition, it is also difficult to distinguish the sequencing errors and PSVs from true SNPs. Take the maize as an example, it has been demonstrated that although millions of SNPs were identified, only a small portion of those polymorphisms could be utilized for the further development of robust and versatile assays [28]. To this end, we explored a simple strategy to develop SNPs from non-model species *P. ginseng* by performing a BLAST homology search against the constructed SCNGs library of Arabidopsis. Accordingly, a total of 22,824 *Panax* ESTs were analyzed and 542 of them showed high similarity to the references of Arabidopsis SCNGs. Forty-five primer pairs were designed from the exon regions of *Panax* SCNGs, of which 16 primer pairs produced clear amplicons of the expected size in *P. ginseng* (Table 1) and ten of which were successfully amplified in *P. quinquefolium* (Table 2). To ensure whether the SNPs were actually retrieved from the orthologous genes, we have analyzed the genetic divergence of all the obtained clones for each putative SCNG. As expected, only a small amount of sites showed single nucleotide variation and almost all of the retrieved SNPs were found at these sites. These attributes suggested that the 16 nuclear genes are likely single copy nuclear gene in *P. ginseng* and *P. quinquefolium*. Through screening DNA polymorphisms of the 16 SCNGs, we successfully identified 160 and 177 SNPs from *P. quinquefolium* and *P. ginseng*, respectively. In addition, the obtained sequences of SCNGs produced alignments ranging from 278 base pair (bp) to 1,339 and 286 to 853 bp in *P. ginseng* and *P. quinquefolium*, respectively. All of these DNA sequences have been submitted to GenBank under the accession numbers of KF529139-KF529528.

### 2.2. Nucleotide Diversity in *P. ginseng* and *P. quinquefolium*

These SNPs can be employed to investigate the molecular phylogenetics, population genetic and molecular breeding of the *Panax* species. For example, although several previous studies have employed allozyme, random amplification polymorphism DNA (RAPD), inter simple sequence repeat (ISSR), amplification fragment length polymorphism (AFLP) and microsatellite techniques to investigate the genetic diversity of *P. ginseng*, these genetic markers are largely from unknown regions of the genome and can not be applied among laboratories that might have less practical value in the further studies [14,15,29]. In this study, we applied these SNPs to evaluate the nucleotide diversity of *P. ginseng* and *P. quinquefolium*. Results from the polymorphic loci of *P. ginseng* revealed that nucleotide diversity ranged from 0.001 to 0.023 for total sites (π_T_) and from 0.000 to 0.017 for nonsynonmous sites (π_Non_), respectively (Table 1). Similarly, nucleotide diversity of *P. quinquefolium* varied from 0.005 to 0.035 for total sites and from 0.000 to 0.079 for nonsynonmous sites, respectively (Table 2). The genetic diversity based on SNP markers has been also reported in some other crop plants. For example, Haudry *et al.* [30] have employed 21 nuclear genes to investigate the genetic diversity of *Triticum turgidum* ssp*. dicoccum* and revealed that this species possessed low genetic diversity (π_T_ = 0.0008). Likewise, low genetic diversity were also found in *Zea may* ssp. *may* (π_T_ = 0.0064) and *Hordeum vulgare* (π_T_ = 0.0031) [31,32]. In comparison with these previous studies, our results showed that although a small amount of individuals of *P. ginseng* and *P. quinquefolium* were investigated respectively, both the two *Panax* species exhibited relatively high level of nucleotide diversity at both total (π_T_ are 0.007 and 0.017 for *P. ginseng* and *P. quinquefolium*, respectively) and nonsynonmous (π_T_ are 0.004 and 0.024 for *P. ginseng* and *P. quinquefolium*, respectively) sites. Notably, we found that *P. quinquefolium* showed relatively higher genetic diversity at both total and nonsynonmous sites in comparison with *P. ginseng*. It indicated that *P. ginseng* might have undergone genetic bottleneck during the domestication process. In addition, Wang *et al.* [33] have developed an amplification refractory mutation system (ARMS)-PCR method and successfully applied it to identify the ginseng cultivars. Here, our results showed that no haplotypes were shared between *P. ginseng* and *P. quinquefolium*. It suggested that these molecular markers could be employed to distinguish the two *Panax* species.

## 3. Experimental Section

### 3.1. Samples and DNA Extraction

SNPs discovery was assessed in samples from 20 individuals of *P. ginseng* and ten individuals of *P. quinquefolium*. The detailed information of the specimens was listed in Table 3. In general, the 20 samples were collected from ten localities and each of them contained two individuals. Similarly, the ten individuals of *P. quinquefolium* were also obtained from two locates. Genomic DNA was extracted from leaves of each individual using a Plant Genomic DNA kit (TianGen, Beijing, China) following the manufacturer’s protocols.

### 3.2. SCNG Library Construction and Primer Design

To obtain SNPs from *P. ginseng* and *P. quinquefolium*, library of SCNGs was constructed based on the database of putative SCNGs (see in the Supplementary File 1). In detail, available references of Arabidopsis were retrieved from GenBank according to the accession number of Duarte *et al.* [34]. Then, all ESTs and genomic sequences of *Panax* were downloaded from GenBank and aligned against the constructed SCNGs library of Arabidopsis using Basic Local Alignment Search Tool (BLAST). For aligned EST sequences that satisfy minimum matched query length of 200 nucleotides and identify of 80% were considered as valid hits. To further identify the gene structures of SCNGs in *P. ginseng*, we blasted these ESTs against the BLASTX of GenBank (http://www.ncbi.nlm.nih.gov/dbEST/index.html) [35]. The exon-intron boundaries of SCNGs were determined by available annotated references. The identified *Panax* ESTs were subjected to design primers using the software Primer Premier 5.0 (Premier Biosoft International, Palo Alto, CA, USA).

### 3.3. PCR, Sequencing and Gene Function Prediction

The designed primer pairs were further employed to amplify the target fragments of *P. ginseng* and *P. quinquefolium*. PCRs were performed using an ABI 2720 Thermocycler (Applied Biosystems, Foster City, CA, USA) in a 30 μL total volume containing: 20–50 ng template DNA, 1× PCR buffer (Mg^2+^ free), 2.5 mM Mg^2+^, 0.6 μM of each primer, 0.2 mM of each dNTP, 1 unit of rTaq polymerase (Takara, Dalian, Liaoning, China). The amplifications were performed under the following conditions: 94 °C for 5 min, 35 cycles of 30 s at 94 °C, 30 s at the annealing temperature (Tables 1 and 2) for each designed specific primer, 90 s at 72 °C, and a final extension of 72 °C for 8 min. All amplified products were separated by electrophoresis on 1.5% agarose gels and purified with the Gel DNA Recovery Kits (Takara) following manufacturer’s instructions and sequenced with the ABI3730 sequencer (Beijing Invitrogen Biotechnology CO., Ltd., Beijing, China). Previous studies have documented that *P. ginseng* and *P. quinquefolium* are tetraploid species [36–38]. To ensure all the SNPs were retrieved from the orthologous genes, we have therefore sequenced more than 10 clones from the same individual for each putative SCNGs and analyzed the genomic divergence of the obtained sequences. To further determine the function of SCNGs, the obtained genomic sequences were searched against the GenBank non-redundant protein database of *Arabidopsis thaliana* using BLASTX [35] with an expected value <10^−7^. The putative functions of these SCNGs are listed in Table 1.

### 3.4. SNP Genotyping and Data Analyses

Obtained DNA sequences of *P. ginseng* and *P. quinquefolium* were subsequently subjected to identify the SNPs. Initial sequence editing and assembly was performed using the ContigExpress (Informax Inc., North Bethesda, MD, USA, 2000). DNA sequence alignment was implemented in ClustalX 1.83 [39] and if necessary edited manually in BioEdit 7.0.1 [40]. To evaluate nucleotide diversity of the two *Panax* species, nucleotide polymorphisms were analyzed using DnaSP version 5 [41], including number of segregating sites (*S*), number of haplotypes (*h*) and haplotype diversity (*H*_d_). In addition, we also surveyed nucleotide diversity π [42] for total and nonsynonymous sites for each locus and the combined dataset separately. The insertions/deletions (indels) were not included in these analyses.

## 4. Conclusions

SNPs are increasingly being used as an ideal molecular marker in both model and non-model species. Here, we explored an approach of development SNPs from the SCNGs of non-model species *P. ginseng* and *P. quinquefolium*. Our results suggested that this strategy could also be applied to develop SNPs in other model or non-model species.

## Figures and Tables

**Table 1.** Nucleotide diversity of the 16 single copy nuclear genes in *Panax ginseng*.

Locus	Primer sequences (5′-3′)	Alignment (bp)		*T*_a_ (°C)	*S*	*h*	*H*_d_	π_T_	π_Non_	Function annotation

		exon	intron							
PGN7	F: CCCAATGCCCCCAGAGTTTT R: AGCGAGGTGCTGCTTGAAGT	441	336	54	21	6	0.779	0.011	0.003	beta-amyrin synthase
PW2	F: AGCACAAGCTCAAGCGTCTC R: CAGTTGGCTGGCATAACACC	63	269	48	5	12	0.947	0.007	0.015	40S ribosomal protein S27
PW8	F: ATAGCTCGTGTAACTGATGG R: TTGAGTGCGGGTGTCTGAAT	119	555	64	30	10	0.926	0.000	0.000	vesicle transport protein
PW16	F: ATTGGTGGAGGGAAGGAACT R: GAGTGGCATGAGCAGTATGT	170	278	52	7	9	0.905	0.009	0.004	prolyl-tRNA synthetase
PW21	F: AAAAGGTTGGCTACGAGTGG R: TACATGATGGGTGGAGGAGA	146	140	64	2	3	0.658	0.003	0.000	photosystem I reaction center subunit N
PW28	F: GGGGTGGGAATTTGGAAGTA R: TGAAGGAGCATCGGAACCAT	155	205	60	15	2	0.526	0.022	0.017	photosystem I reaction center subunit H-2
PZ7	F: ACCTGGTTCGCTGCTATTCC R: CAAGCATTGGTTCCCTCTGG	97	304	52	2	3	0.468	0.001	0.000	PGR5-like protein 1A
PZ12	F: GAGCGTTCTCAAATGCGGTAG R: CTTAGCCTCAAACTGGTCGG	118	830	54	1	2	0.100	0.001	0.000	60S ribosomal protein
PZ15	F: TGAACAGGCATTATTACTCG R: ACTCATCCTCCTCTTGAACG	105	653	48	0	1	0.000	0.000	0.000	26S proteasome non-ATPase
PZ14	F: CTTTGTTTCTCCTCCTCCAG R: GGATTTCCAGAGCAACCTTT	178	667	54	11	4	0.621	0.007	0.000	diacylglycerol kinase 1
PZ10	F: CTATGATGGGGTCTGGAGGG R: AGCAGTGATGGTGGATGAGG	305	445	62	32	8	0.853	0.023	0.013	glycine decarboxylase
PZ13	F: AGCAGCCGAGTATGAAACCC R: CCTCAGGTAAACGATAACCG	174	845	56	0	1	0.000	0.000	0.000	signal peptidase complex subunit 3B
PZ1	F: CACTACCCCGTTCTTTTCCG R: CCTTTTGTTCCTCAACCACC	372	967	60	25	6	0.768	0.010	0.009	glycine decarboxylase P-protein
PZ4	F: TGTTGACCATCTACTCACCCAG R: CCTTCACGCATTCCCACAAT	207	426	48	7	8	0.895	0.006	0.000	hypothetical protein
PZ5	F: TGACGGACTTGACCTAACAT R: CTTCAGATACAGCCCACAGC	171	707	56	12	4	0.621	0.007	0.000	ABC transporter F family member 3-like
PZ8	F: GGGAAGGAAAAGTTGCTCTG R: TATTCGTGTTGGGGCATCTG	196	545	60	7	4	0.779	0.005	0.000	hypothetical protein

*T*_a_, annealing temperature; *S*, number of segregating sites; *h*, number of haplotypes; *H*_d_, haplotype diversity; π_T_, nucleotide diversity for total sites; π_Non_, nucleotide diversity for nonsynonymous sites.

**Table 2.** Nucleotide diversity of the ten single copy nuclear genes in *Panax quinquefolium*.

Locus	Alignment (bp)		*T*_a_ (°C)	*S*	*h*	*H*_d_	π_T_	π_Non_

	exon	intron						
PGN7	441	338	54	23	7	0.964	0.010	0.001
PW2	60	266	48	15	8	0.956	0.027	0.079
PW16	153	290	40	27	9	0.978	0.035	0.033
PW21	152	134	60	16	2	0.556	0.031	0.047
PZ7	98	290	52	11	4	0.733	0.016	0.015
PZ15	96	665	48	0	1	0.000	0.000	0.000
PZ14	178	675	49	9	5	0.800	0.005	0.004
PZ10	302	445	60	26	2	0.556	0.019	0.021
PZ5	204	516	46	26	7	0.911	0.020	0.040
PZ8	138	477	60	7	4	0.822	0.006	0.000

*T*_a_, annealing temperature; *S*, number of segregating sites; *h*, number of haplotypes; *H*_d_, haplotype diversity; π_T_, nucleotide diversity for total sites; π_Non_, nucleotide diversity for nonsynonymous sites.

**Table 3.** Details of localities sampled from the field in this study and number of individual sequenced for each locus in each locality.

Species name	Locality	Country	Latitude/longitude	Elevation (meter)	Number of individuals	Sampling date	Voucher specimens
*P. ginseng*	TQL	China	43°36′129″N 129°35′807″E	469	2	9/2011	NENU20110902001
	WHL	China	43°30′181″N 127°54′193″E	551	2	9/2011	NENU20110903001
	FS	China	42°24′216″N 127°12′186″E	589	2	7/2011	NENU20110720001
	JY	China	42°23′197″N 126°48′490″E	612	2	7/2011	NENU20110713001
	XJD	China	42°20′870″N 128°44′449″E	845	2	9/2011	NENU20110902002
	CB	China	41°39′442″N 127°35′229″E	936	2	9/2011	NENU20110802001
	LJ	China	41°48′432″N 126°55′530″E	663	2	8/2011	NENU20110801001
	BT	China	41°18′492″N 125°49′954″E	369	2	7/2011	NENU20110718006
	SZ	China	40°45′595″N 125°20′863″E	375	2	7/2011	NENU20110718001
	GL	China	41°25′121″N 128°12′296″E	765	2	9/2012	NENU20130929001

*P. quinquefolium*	WS	USA	n.a.	n.a.	5	9/2012	n.a
	JY	China	42°23′197″N 126°48′490″E	612	5	7/2011	NENU20110713009

n.a., no available data.

## References

[1] Agarwal M., Shrivastava N., Padh H. (2008). Advances in molecular marker techniques and their applications in plant sciences. Plant Cell Report.

[2] Botstein D., White R.L., Skolnick M., Davis R.W. (1980). Construction of a genetic linkage map in man using restriction fragment length polymorphisms. Am. J. Hum. Genet.

[3] Williams J.G.K., Kubelik A.R., Livak K.J., Rafalski J.A., Tingey S.V. (1991). DNA polymorphisms amplified by arbitrary primers areusefll as genetic markers. Nucleic Acids Res.

[4] Vos P., Hogers R., Bleeker M., Reijans M., van de Lee T., Hornes M., Frijters A., Pot J., Peleman J., Kuiper M., Zabeau M. (1995). AFLP: A new technique for DNA fingerprinting. Nucleic Acids Res.

[5] Dong Z., Wang H., Dong Y., Wang Y., Liu W., Miao G.J., Lin X.Y., Wang D.Q., Liu B. (2013). Extensive Microsatellite Variation in Rice Induced by Introgression from Wild Rice (*Zizania latifolia* Griseb.). PLoS One.

[6] Kalia R.K., Rai M.K., Kalia S., Singh R., Dhawan A.K. (2011). Microsatellite markers: An overview of the recent progress in plants. Euphytica.

[7] Varshney R.K., Graner A., Sorrells M.E. (2005). Genic microsatellite markers in plants: Features and applications. Trends Biotechnol.

[8] Clemento A.J., Abadia-Cardoso A., Starks H.A., Garza J.C. (2011). Discovery and characterization of single nucleotide polymorphisms in Chinook salmon *Oncorhynchus tshawytscha*. Mol. Ecol. Resources.

[9] Helyar S.J., Hemmer-Hansen J., Bekkevold D., Taylor M.I., Ogden R., Limborg M.T., Cariani A., Maes G.E., Diopere E., Carvalho G.R. (2011). Application of SNPs for population genetics of nonmodel organisms: New opportunities and challenges. Mol. Ecol. Resources.

[10] Geraldes A., Pang J., Thiessen N., Cezard T., Moore R., Zhao Y., Tam A., Wang S., Friedmann I., Seeb J.E. (2011). SNP discovery in black cottonwood (*Populus trichocarpa*) by population transcriptome resequencing. Mol. Ecol. Resources.

[11] Howe G.T., Yu J., Knaus B., Cronn R., Kolpak S., Dolan P., Lorenz W.W., Dean J.F. (2013). A SNP resource for Douglas-fir: De novo transcriptome assembly and SNP detection and validation. BMC Genomics.

[12] Wen J., Zimmer E.A. (1996). Phylogeny and biogeography of *Panax* L. (the ginseng genus, Araliaceae): Inferences from ITS sequences of nuclear ribosomal DNA. Mol. Phylogenet. Evol.

[13] Lee C., Wen J. (2004). Phylogeny of *Panax* using chloroplast *trnC–trnD* intergenic region and the utility of *trnC–trnD* in interspecific studies of plants. Mol. Phylogenet. Evol.

[14] Reunova G.D., Kats I.L., Muzarok T.I., Zhuravlev Y.N. (2010). Polymorphism of RAPD, I.S.S.R. and AFLP markers of the Panax ginseng C.A. Meyer (Araliaceae) genome. Russ. J. Genet.

[15] Zhuravlev Y.N., Reunova G.D., Kats I.L., Muzarok T.I., Bondar A.A. (2010). Molecular variation of wild *Panax ginseng* C.A. Meyer (Araliaceae) by AFLP markers. Chin. Med.

[16] Song C.S., Xu R.Z., Zhang Q.H. (1989). Rare and Endangered Plants in China.

[17] Sun J., Chen P. (2011). Differentiation of *Panax quinquefolius* grown in the USA and China using LC/MS-based chromatographic fingerprinting and chemometric approaches. Anal. Bioanal. Chem.

[18] Benson D.A., Karsch-Mizrachi I., Lipman D.J., Ostell J., Sayers E.W. (2010). GenBand. Nucleic Acids Res.

[19] Arai-Kichise Y., Shiwa Y., Nagasaki H., Ebana K., Yoshikawa H., Yano M., Wakasa K. (2011). Discovery of genome-wide DNA polymorphisms in a landrace cultivar of japonica rice by whole-genome sequencing. Plant Cell Physiol.

[20] Cao J., Schneeberger K., Ossowski S., Günther T., Bender S., Fitz J., Koenig D., Lanz C., Stegle O., Lippert C. (2011). Whole-genome sequencing of multiple *Arabidopsis thaliana* populations. Nat. Genet.

[21] Stölting K.N., Nipper R., Lindtke D., Caseys C., Waeber S., Castiglione S., Lexer C. (2013). Genomic scan for single nucleotide polymorphisms reveals patterns of divergence and gene flow between ecologically divergent species. Mol. Ecol.

[22] Subbaiyan G.K., Waters D.L., Katiyar S.K., Sadananda A.R., Vaddadi S., Henry R.J. (2012). Genome-wide DNA polymorphisms in elite indica rice inbreds discovered by whole genome sequencing. Plant Biotechnol. J.

[23] Seeb J.E., Carvalho G., Hauser L., Naish K., Roberts S., Seeb L.W. (2011). Single-nucleotide polymorphism (SNP) discovery and applications of SNP genotyping in nonmodel organisms. Mol. Ecol. Resources.

[24] Ekblom R., Galindo J. (2010). Applications of next generation sequencing in molecular ecology of non-model organisms. Heredity.

[25] O’Neil S., Dzurisin J.D., Carmichael R.D., Lobo N.F., Emrich S.J., Hellmann J.J. (2011). Population-level transcriptome sequencing of nonmodel organisms *Erynnis propertius* and *Papilio zelicaon*. BMC Genomics.

[26] Parchman T., Geist K., Grahnen J., Benkman C., Buerkle C.A. (2011). Transcriptome sequencing in an ecologically important tree species: Assembly, annotation, and marker discovery. BMC Genomics.

[27] Sánchez C., Smith T., Wiedmann R., Vallejo R., Salem M., Yao J., Rexroad C. (2009). Single nucleotide polymorphism discovery in rainbow trout by deep sequencing of a reduced representation library. BMC Genomics.

[28] Mammadov J., Chen W., Mingus J., Thompson S., Kumpatla S. (2012). Development of versatile gene-based SNP assays in maize (*Zea mays* L.). Mol. Breeding.

[29] Koren O.G., Potenko V.V., Zhuravlev Y.N. (2003). Inheritance and variation of allozymes in Panax ginseng CA Meyer (Araliaceae). Int. J. Plant Sci.

[30] Haudry A., Cenci A., Ravel C., Bataillon T., Brunel D., Poncet C., Hochu I., Poirier S., Santoni S., Glémin S. (2007). Grinding up wheat: A massive loss of nucleotide diversity since domestication. Mol. Biol. Evol.

[31] Wright S.I., Bi I.V., Schroeder S.G., Yamasaki M., Doebley J.F., McMullen M.D., Gaut B.S. (2005). The Effects of Artificial Selection on the Maize Genome. Science.

[32] Kilian B., Özkan H., Kohl J., Haeseler A., Barale F., Deusch O., Brandolini A., Yucel C., Martin W., Salamini F. (2006). Haplotype structure at seven barley genes: Relevance to gene pool bottlenecks, phylogeny of ear type and site of barley domestication. Mol. Genet. Genomics.

[33] Wang H., Sun W., Kwon W.S., Jin H., Yang D.C. (2010). A PCR-based SNP marker for specific authentication of Korean ginseng (*Panax ginseng*) cultivar “Chunpoong”. Mol. Biol. Reports.

[34] Duarte J.M., Wall P.K., Edger P.P., Landherr L.L., Ma H., Pires J.C., Leebens-Mack J. (2010). Identification of shared single copy nuclear genes in Arabidopsis, Populus, Vitis and Oryza and their phylogenetic utility across various taxonomic levels. BMC Evol. Biol.

[35] Altschul S.F., Madden T.L., Schäffer A.A., Zhang J., Zhang Z., Miller W., Lipman D.J. (1997). Gapped BLAST and PSI-BLAST: A new generation of protein database search programs. Nucleic Acids Res.

[36] Hu S. Biological and Cytological Foundation for Better Ginseng to More People.

[37] Grushvitskii I.V. (1961). Zhenshen: Voprosy Biologii (Ginseng: Problems in Biology).

[38] Bulgakov V.P., Lauve L.S., Tchernoded G.K., Khodakovskaya M.V., Zhuravlev Y.N. (2000). Chromosome Variation in Ginseng Cells Transformed with the *rolC* Plant Oncogene. Russ. J. Genet.

[39] Thompson J.D., Gibson T.J., Plewniak F., Heanmougin F., Higgins D.G. (1997). The CLUSTAL_X windows interface: Flexible strategies for multiple sequence alignment aided by quality analysis tools. Nucleic Acids Res.

[40] Hall T.A. (1999). BioEdit: A user-friendly biological sequence alignment editor and analysis program for Windows 95/98/NT. Nucleic Acids Symp. Ser.

[41] Rozas J., Sánchez-Delbarrio J.C., Messeguer X., Rozas R. (2003). DnaSP, DNA polymorphism analyses by the coalescent and other methods. Bioinformatics.

[42] Tajima F. (1983). Evolutionary relationship of DNA sequences in finite populations. Genetics.

